# Glaucoma, Pseudoexfoliation and Hearing Loss: A Systematic Literature Review

**DOI:** 10.3390/jcm13051379

**Published:** 2024-02-28

**Authors:** Laura Antonia Meliante, Giulia Piccotti, Lucia Tanga, Sara Giammaria, Gianluca Manni, Giulia Coco

**Affiliations:** 1 Department of Clinical Sciences and Translational Medicine, University of Rome Tor Vergata, 00133 Rome, Italy; lauraantonia.meliante@ptvonline.it (L.A.M.); giulia.piccotti@ptvonline.it (G.P.); giulia.coco@uniroma2.it (G.C.); 2 IRCCS—Fondazione Bietti, 00184 Rome, Italy; lucia.tanga@fondazionebietti.it (L.T.); sara.giammaria@fondazionebietti.it (S.G.)

**Keywords:** glaucoma, hearing loss, pseudoexfoliation, POAG, NTG

## Abstract

**Purpose:** To investigate the relationship between glaucoma, pseudoexfoliation and hearing loss (HL). **Methods**: A systematic literature search following PRISMA guidelines was conducted using the PubMed, Embase, Scopus and Cochrane databases from 1995 up to 28 August 2023. **Results**: Thirty studies out of the 520 records screened met the inclusion criteria and were included. Most articles (n = 20) analysed the association between pseudoexfoliation syndrome (XFS) and HL, showing XFS patients to have higher prevalence of sensorineural hearing loss (SNHL) at both speech frequencies (0.25, 0.5, 1 and 2 kHz), and higher frequencies (4 and 8 kHz) compared to controls in most cases. No significant differences in prevalence or level of HL between XFS and pseudoexfoliative glaucoma (XFG) were detected in most studies. Eight articles analysed the relationship between primary open-angle glaucoma (POAG) and HL. Overall, a positive association between the two conditions was highlighted across all studies except for two cases. Similarly, articles focusing on NTG and HL (n = 4) showed a positive association in most cases. The role of autoimmunity and, in particular, the presence of antiphosphatidylserine antibodies (APSA) in patients with NTG and HL suggested an underlying autoimmune or vascular mechanism contributing to their pathogenesis. Only one study analysed the relationship between angle-closure glaucoma (ACG) and HL, showing higher incidence of ACG in patients with SNHL compared to normal hearing controls. **Conclusions**: Most studies detected an association between XFS and HL as well as POAG/NTG/ACG and HL, suggesting the presence of a similar pathophysiology of neurodegeneration. However, given the strength of the association of XFS with HL, it remains unclear whether the presence of XFG is further associated with SNHL. Further research specifically targeted to assess the correlation between glaucoma, XFS and HL is warranted to provide a more comprehensive understanding of this association.

## 1. Introduction

The term glaucoma refers to a group of progressive optic neuropathies that determine visual field defects and can potentially result in vision loss; it represents the leading cause of irreversible blindness globally, with prevalence increasing with age and reaching 10% in individuals over 90 years old [1]. Degeneration of the optic nerve, thinning of the retinal fibres and loss of retinal ganglion cells (RGCs) are common manifestations among all glaucoma subjects [2,3]. Although elevated intraocular pressure (IOP) is the major and only actually modifiable risk factor, many individuals with glaucoma experience disease progression despite having normal IOP levels [4]. This suggests that factors other than intraocular pressure, such as genetic factors, structural vulnerability and vascular abnormalities, may play a role in the development of the condition.

Many studies promote the role of vascular abnormalities in glaucomatous damage [4,5]. According to this hypothesis, reduced perfusion pressure, deficient vascular autoregulation and loss of neurovascular coupling can lead to damage of the optic nerve head [4,5,6,7,8,9,10,11]. Abnormality in vascular function also influences the secretion and drainage of aqueous humour, consequently increasing IOP and contributing to the pathogenesis and progression of glaucoma [5].

Neurodegeneration also plays a role. Glaucomatous degeneration has been observed along the entire optical pathway, starting from the optic papilla and reaching lateral geniculate nucleus and visual cortex of the brain. The spread of disease between communicating neurons has been named “transsynaptic degeneration” and is a well-known process in neurological diseases like Alzheimer’s and amyotrophic lateral sclerosis, and has also been described in experimental primate and human glaucoma [12,13,14]. Mechanisms involved in central visual system damage in glaucoma subjects include oxidative injury and glutamate toxicity, as seen in other neurodegenerative diseases [15].

Among causes of secondary glaucoma, pseudoexfoliation syndrome (XSF) is a systemic, age-related disorder characterized by the deposition of extracellular fibrillar material in different organs, which confers a ten times higher risk of developing glaucoma [16,17]. Pseudoexfoliative material accumulates mainly on the iris, anterior lens capsule, ciliary body and trabecular meshwork, resulting in aqueous outflow obstruction, raised intraocular pressure (IOP) and secondary open angle glaucoma [18]. As for primary glaucoma, vascular abnormalities and neurodegeneration potentially play a role through deposition of fibrillar material in the vessel walls and abnormal fibrillar material deposition, similar to the Aβ-deposits in Alzheimer’s disease, Lewy bodies in Parkinson’s disease and drusen in age-related macular degeneration, with higher incidence of neurodegenerative disorders in patients with XFS [19,20,21,22,23,24,25,26,27,28].

Overall, people with glaucoma have been suggested to be more vulnerable to central nervous system (CNS) neural decline; therefore they may also be more likely to exhibit functional deficits beyond the visual system. In primary open angle glaucoma (POAG) patients, temporal processing deficits, consistent with central processing abnormalities, have been demonstrated to impact both the visual and auditory domains, thus suggesting a correlation between glaucoma and auditory impairment [29].

Studies on glaucoma and sensorineural hearing loss (SNHL) have shown that both diseases share a similar pathophysiology of neurodegeneration [30,31]. SNHL refers to hearing impairment secondary to cochlear abnormalities and/or damage to the cochlear nerve or to the central auditory pathways [31]. The most common form of SNHL is age-related hearing loss, characterized by a gradual and symmetrical decrease in hearing sensitivity, particularly at higher frequencies, affecting over 60% of individuals aged sixty-five and above [32,33]. SNHL is a complex condition influenced by various factors, including gender, ethnicity, noise exposure, ototoxic medications, lifestyle choices, comorbidities and genetic predisposition [34]. However, SNHL has been associated with higher prevalence of neurodegenerative or neurological diseases like Alzheimer’s, cognitive impairment, or general dementia, through both common and causal mechanisms [35]. Specifically, microvascular alterations may induce ischemic injury to the inner ear structures, resulting in insufficient cochlear blood flow; similarly, small vessel disease in the brain may lead to reduced cognitive function due to decreased blood supply [36,37]. Additionally, studies have pointed out abnormalities in the stria vascularis of subjects with SNHL, confirming the vascular component of this condition [38,39].

The above-mentioned pathogenetic mechanisms suggest a potential link between SNHL and glaucoma. The aim of this review was therefore to investigate current evidence for the association between glaucoma and SNHL.

## 2. Materials and Methods

A systematic literature review (SLR, without registration) was conducted to investigate the association between glaucoma and hearing loss (HL); the research of publications of interest was conducted by querying the PubMed, Embase, Scopus and Cochrane databases. Each database was searched for articles dating from 1995 until 28 August 2023. Search terms included a combination of database-specific controlled vocabulary terms and free-text terms relating to glaucoma and hearing loss (POAG OR glaucoma OR XFG or “pigmentary glaucoma”) AND (deaf* OR “hearing loss”). We selected only full articles, written in English, which included a study population of adult subjects (18 years old or over).

**Figure 1:**
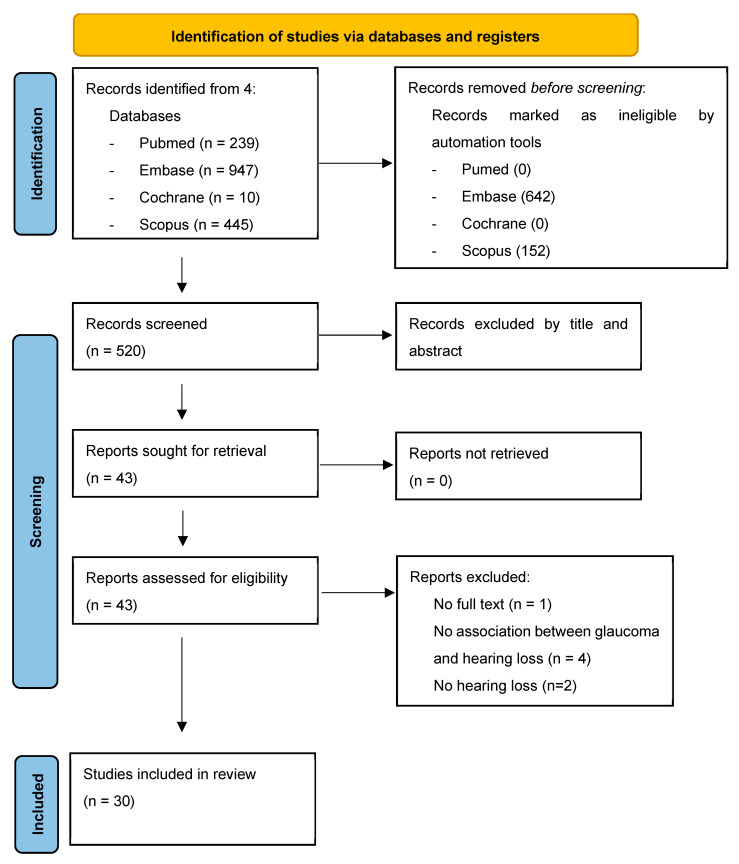
PRISMA diagram of literature search.

All articles were identified by searching the three databases and were imported into Mendeley for screening. After deduplication, two screening rounds were performed, as described in Figure 1.

In the first round, two reviewers (L.A.M. and G.P.) evaluated, in duplicate, titles and abstracts in terms of relevance for both glaucoma and hearing loss. Articles were selected based on the exclusion criteria reported in Figure 1. In the second round, the full texts of the articles included during the first round were retrieved and re-assessed for eligibility. Possible discordance during study selections was discussed with a third reviewer (G.C.) to reach a consensus.

A detailed flow chart describing the study inclusion and exclusion process is presented in Figure 1.

### 2.1. Sensorineural Hearing Loss Assessment

Complete audiometric evaluation is the gold standard for assessing hearing loss. In clinical practice, pure tone audiometry (PTA) and tympanometry are widely used tests [40].

#### 2.1.1. Pure-Tone Audiometry

PTA is a behavioural test used to measure pure-tone hearing thresholds (PTTs) at 0.25–8 kHz for each ear. PTTs indicate the softest sound audible to an individual at least 50% of the times. Measurements involve both the peripheral and the central auditory system; both air and bone conduction pathways can be detected, with relative thresholds plotted on a graph.

PTA at 0.5, 1 and 2 kHz (PTA_512_) is important for understanding speech in quiet conditions, while PTA at 3, 4, 6 and 8 kHz (PTA_3468_) is important for distinguishing consonants that have high frequency acoustic energy that contribute to the understanding of speech in noisy environments.

Normal hearing refers to a hearing threshold of 0–25 dB, and degrees of hearing loss can be categorized at each tested frequency in mild hearing impairment (26–40 dB), moderate hearing impairment (41–60 dB), severe hearing impairment (61–80 dB) and profound hearing impairment (>80 dB) [41].

In SNHL, both air and bone conduction curves worsen, with no air–bone gap [40]. Conductive HL has normal bone conduction and abnormal air conduction with an air–bone gap of at least 10 dB, while mixed forms have both conductive and sensorineural components, with abnormal PTT for both air and bone conduction and an air–bone gap of more than 10 dB.

#### 2.1.2. Tympanometry

Tympanometry tests middle ear function and tympanic membrane mobility by creating variations of air pressure in the ear canal. A tone of 226 Hz is generated by a tympanometer placed in the ear canal, where the sound strikes the tympanic membrane. Some of this sound is reflected and picked up by the instrument. Middle ear problems often result in stiffening of the middle ear, which causes more of the sound to be reflected back [42]. The peak value of the compliance (mobility) curve is recorded. SNHL is characterized by a normal type A curve, while conductive or mixed HL shows abnormal results (type B-flat or type C-curve with admittance peak shifted left or negative). Because tympanometry is not a hearing test, it is always performed in conjunction with PTA.

The acoustic stapedial reflex can also be tested with tympanometry, since the contraction of the stapedius muscle stiffens the middle ear, thus decreasing middle ear admittance. The lowest intensity of sound that triggers the reflex is the acoustic reflex threshold [40].

#### 2.1.3. Otoacoustic Emissions

Otoacoustic emissions (OAEs) are sounds generated from the cochlea and transmitted across the middle ear to the external ear canal, where they can be recorded [43]. The production of an OAE is a marker for inner ear health and a simple way to screen for hearing loss [44]. OAEs are divided into spontaneous otoacoustic emissions (SOAEs), occurring without external stimuli, and evoked OAEs (EOAEs), which are measured after an acoustic stimulus. Distortion product otoacoustic emissions (DPOEs) measure cochlear activity.

#### 2.1.4. Auditory Brainstem Response

The auditory brainstem response (ABR) tests for functional changes in the retrocochlear auditory pathway, from the auditory nerve to the mesencephalon. ABR is based on auditory evoked potential and tests for synchronous neural function, and it is usually performed to estimate hearing thresholds in individuals who are unable to undergo traditional audiometry [45]. Pathological ABR morphology should prompt magnetic resonance imaging (MRI) test.

## 3. Results

Thirty studies out of the 520 records screened met the inclusion criteria and were considered for this review. Most of them (n = 20) discussed the relationship between pseudoexfoliation syndrome (XFS) and HL. Study characteristics and demographic information of each article, as well as results on XFS, pseudoexfoliative glaucoma (XFG), POAG, normal tension glaucoma (NTG) and angle closure glaucoma (ACG), are summarized in Table 1, Table 2 and Table 3.

### 3.1. Pseudoexfoliation Syndrome, Pseudoexfoliative Glaucoma and Hearing Loss

Pseudoexfoliation is a systemic, age-related disorder characterized by the deposition of extracellular fibrillar material in different parts of the body. Deposition of this fibrillar material has been associated with several diseases, like abdominal aortic aneurism, peripheral vascular disease, renal artery stenosis, obstructive sleep apnoea, Alzheimer’s disease and also sensorineural hearing loss and glaucoma [27,46,47,48,49,50,51,52,53]. Pseudoexfoliation syndrome is usually diagnosed by the observation of pseudoexfoliative material deposition in different parts of the anterior segment of the eye, specifically on the anterior lens capsule.

The link between XFS and glaucoma lie in the fact that the deposition of this material in the trabecular meshwork can result in aqueous outflow obstruction, raised IOP and secondary open angle glaucoma, known as pseudoexfoliative glaucoma [54,55,56].

Similarly, there is a link between XFS and SNHL. The first pathogenetic hypothesis is a disfunction of hearing mechanoreceptors due to the deposition of fibrillar material in either or both the tectorial and basilar membranes, impairing the transmission of vibrating energy to the sensory hair cells, hence the conversion of the vibration energy to bioelectric energy [47,57]. An alternative mechanism is vascular compromission due to the deposition of PEX fibrils in the vessel walls [23,24,25,48]. This may cause a dysfunction in the metabolism of the stria vascularis, which regulates ion balance of endolymph and perilymph and reduces the vascularization of the inner ear. Malnutrition of the cochlear basal cells, which are more susceptible to ischemia, can lead to an SNHL in which higher frequencies are predominantly affected [58].

The association with cardiovascular disorders and the evidence of decreased blood flow velocity, increased vascular resistance in middle cerebral arteries and impaired systemic endothelial cell function in patients with pseudoexfoliation support this theory [59,60]. It is indeed plausible that both these pathogenic mechanisms work together in the injury onset.

The correlation between XFS, XFG and SNHL has been extensively investigated.

#### 3.1.1. Pseudoexfoliation Syndrome

Twenty articles in our SRL analysed the relationship between XFS and HL. XFS patients were frequently reported to have higher frequencies of SNHL compared to controls with PTA testing [61].

Several studies detected increased PTA thresholds at speech frequencies (0.25, 0.5, 1 and 2 kHz) [62,63,64,65]. Singham et al. found higher prevalence of hearing loss in XFS subjects and significantly higher hearing thresholds at 0.5 and 1 kHz compared to controls (*p* < 0.05) [63]. Later, Temporale et al. (2016), detected abnormal PTA at 2 kHz in 87% of XFS patients compared to 64.3% of controls (*p* = 0.008), and Lee et al. (2017) found XFS patients to have moderate to severe SNHL in 64% of cases (vs. 40.3% in controls) with an average hearing threshold of 47.93 dB, corresponding to the moderate hearing loss category according to the ISO 1964 and significantly decreased PTA at 1 and 2 kHz after age–sex adjustment [62]. Also, Tekin et al. (2020) detected SNHL in 73.8% of XFS patients compared to 58.8% of controls and XFS patients to have significantly higher hearing thresholds in air conductance at 0.25 kHz (35.91 dB vs. 26.53 dB in controls, *p* = 0.001), 0.5 kHz (34.09 dB vs. 23.89 dB; *p* = 0.001), 1 kHz (33.33 dB vs. 22.92 dB; *p* = 0.002), 2 kHz (35.45 dB vs. 27.08; *p* = 0.029) and 4 kHz (52.12 dB vs. 43.47 dB; *p* = 0.036), with, however, no significant differences at the 6 and 8 kHz frequencies. Similar results were obtained for bone conductance [64].

Increased PTA thresholds in XFS subjects were also noted at higher frequencies (4 and 8 kHZ) [66,67]. Papadopoulus et al. showed higher PTA thresholds in XFS at 4 kHz (56.60 ± 17.39 vs. 44.32 ± 18.85 dB in controls, *p* = 0.004) and 8 kHz (73.40 ± 18.71 vs. 58.86 ± 25.91 dB in controls, *p* = 0.001), with the greatest difference at 8 kHz [67]. Furthermore, Bilgeç et al. detected sensorineural decline at 4 and 8 kHz in XFS compared to controls, without, however, controlling for age that seemed different between groups (66.12 ± 5.64 vs. 61.70 ± 8.46 years in XFS and controls, respectively) [66].

Some authors also detected simultaneous impairment at all tested frequencies [67,68]. Ozkan et al., in a prospective case-control study on 75 XFS patients and 75 age–sex matched controls noted SNHL at speech frequencies (mean value of 0.5, 1 and 2 kHz) in 69% of XFS patients vs. 52% of controls (*p* = 0.03) with a significantly higher mean threshold value (33.6 ± 18.5 dB vs. 28.4 ± 15.0 dB, *p* = 0.01) [69]. XFS patients also showed significantly increased threshold levels at 2, 4 and 8 kHz (all *p* < 0.04). Ozturk et al. (2008) also detected SNHL more frequently in XFS patients compared to controls (79.4% vs. 26.3%, *p* < 0.01) with involvement of high frequencies in 58% of cases and of all frequencies in 40% [69]. Furtermore, Papadopoulos et al. found higher prevalence of PTA hearing loss in XFS patients at both low frequencies (0.25 & 0.5 Hz—57% vs. 41%, *p* = 0.07), medium frequencies (1 & 2 kHz—81% vs. 59%, *p* = 0.007) and high frequencies (4 & 8 kHz—98% vs. 86%, *p* = 0.007), with more severe hearing loss noted at 4 and 8 Hz [70].

When other hearing tests were used, Temporale et al. identified the stapedius reflex in a greater proportion of XFS patients, while no differences were noted in DPOAEs and ABR compared to controls [62].

In contrast with the above-mentioned results, Muhafiz et al. did not find any significant difference in hearing loss prevalence and hearing thresholds at 0.25, 0.5, 1, 2, 4, 6 and 8 kHz between XFS and controls (*p* > 0.05) [71]. Additionally, Tryggvason et al. examined hearing loss in 158 patients with XFS/XFG, 95 POAG and 123 controls, and no significant differences were found at either low or high frequencies between groups after controlling for age, sex and possible confounders [72]. The only possible exception was for PTA at 0.5, 1 and 2 kHz in persons of 80+ years with XFS/XFG (*p* = 0.049). However, due to the small numbers included, the power to detect true differences was low. Hearing loss was instead associated with male gender and older age [72].

Overall, no correlation between the worse eye and the worse ear was found, nor any correlation between the side of the eye with PEX and the ear with hearing loss, suggesting that XFS may be a bilateral disease with asymmetrical presentation [57,73]. Also, no correlation between plasma homocysteine levels and hearing loss in XFS was reported [69].

Details on the above studies are summarized in Table 1.

**Table 1:** Pseudoexfoliation syndrome, pseudoexfoliative glaucoma and hearing loss.

First Author (Year)	Glaucoma Type	Study Design	N Cases (n Ears)	Mean Age Patients (Mean ± SD)	% Males Patients	Outcome Measured	Main Findings
			N Controls (n Ears)	Mean Age Controls (Mean ± SD)	% Males Controls		
Bilgeç M.D. (2021) [66]	XFS	case-control	16 (32)	66.12 ± 5.64	62.5%	PTA bone conduction 0.5, 1, 2 and 4 KHz, air- conduction at 0.5–8 kHz, tympanometry, bithermal caloric test, VEMP	SNHL at 4 and 8 kHz in XFS patients compared to control group (*p* < 0.05)
			17 (34)	61.70 ± 8.46	70.6		
Cahill M. (2002) [46]	XFS, XFG	cross-sectional	69 (137)	75.5	56.5%	1, 2 and 3 kHz, ISO 7029	73.7% of XFS patients had a higher threshold than ISO 7029 median AAHL1,2,3; no significant difference between XFS and XFG (*p* = 0.58 for male patients and *p* = 0.60 for female patients).
			/	/	/		
Detorakis E.T. (2008) [74]	XFS, XFG	prospective	54 (108)	68.11 ± 2.11	61.11%	Audiometry, bone and air conduction, PTA 0.25, 1, 2, 3 and 8 kHz; tympanometry	Bone and air audiometric thresholds were significantly increased in XFS patients for 3 kHz (*p* = 0.04 and *p* = 0.03) and 8 kHz (*p* = 0.02 and *p* = 0.04) but not for 0.25 kHz, 1 kHz and 2 kHz (*p* ≥ 0.2). Tympanometric peak values were significantly lower in SG compared with CG (*p* = 0.04)
			48 (96)	67.14 ± 1.30	62.06%		
Gülyeşil F.F. (2023) [75]	POAG XFG	case-control	24 (24) POAG 22 (22)XFG	64.50 ± 7 POAG 66.90 ± 4.51 XFG	50% POAG 50% XFG	PTA 0.25, 0.5, 1, 2, 4, 8, 10 kHz	POAG: higher hearing thresholds at 0.5 (*p* = 0.011) and 1 kHz (*p* = 0.003). XFG: higher hearing thresholds at 0.25 (*p* = 0.009), 0.5 (*p* = 0.009), 1 (*p* = 0.001), 2 (*p* = 0.005), 4 (*p* = 0.001), 8 (*p* = 0.010) and 10 kHz (*p* = 0.009) XFG group: higher hearing threshold at 8 kHz than the POAG group (*p* = 0.002).
			21 (21)	64.38 ± 4.36	57.1%		
Lee S.Y. (2017) [65]	XFS	case control	28 (56)	73.6 ± 7.9	28%	PTA 0.5, 1, 2, 4 and 6 kHz; ISO 1964	statistically significant decrease at 1 and 2 kHz in XFS group; moderate to severe SNHL in 64% of XFS (*p* = 0.023).
			277 (554)	64.7 ± 9.8	34%		
Muhafiz E. (2021) [71]	XFS	case-control	36 (n/a)	71.38 ± 6.88	66.7%	PTA 0.25, 0.5, 1, 2, 4, 6 and 8 kHz	no correlation between XFS and HL: no difference in all evaluated frequencies between XFS and controls (*p* > 0.05).
			39 (n/a)	68.92 ± 8.74	56.4%		
Ozkan B. A. (2006) [69]	XFS, XFG	case-control	75 (150)	68.25 ± 7.41	60%	PTA 0.5, 1, 2, 4 and 8 kHz, plasma homocysteinemia	HL at speech frequencies (0.5, 1, 2 kHz) was higher in XFS (69%) than controls (52%) (*p* = 0.01). Homocysteine levels in patients with XFS and HL were not significatively different from XFS without HL (*p* = 0.5).
			75 (150)	66.76 ± 8.15	52%		
Ozturk F. (2008) [68]	XFS	case-control	63 (126)	68.4 ± 10.3	55.6%	PTA (air and bone conduction), 0.5, 1, 2, 3, 4 and 6 kHz, laterality of HL	HL in 79% of XFS vs. 26% in the control group; Among XFG patients with HL, 96% showed bilateral HL and 58% showed HL at higher frequencies (2–6 kHz).
			38 (76)	65.2 ± 12.3	47.4%		
Paliobei V.P. (2011) [76]	XFG, POAG	prospective	85 (170) POAG 110 (220) XFG	67.4 ± 4.6 XFG 64.8 ± 6.5 POAG	44.7% POAG 58.2% XFG	PTA 0.25, 0.5, 1, 2, 4 and 8 kHz, tympanometry, stapedial reflex test, ABRs, DPOAEs, ISO 7029	HL was more prevalent in POAG and XFG compared to the ISO 7029 (XFG > POAG); DPOAs amplitudes at high frequencies were reduced in both POAG and XFG. Pathologic ABR 4 times higher in XFG than POAG (*p* < 0.001).
			/	/	/		
Papadopoulos T.A. (2010) [67]	XFS	case-control	47 (94)	74.7 ± 6.78	48.9%	PTA 0.25, 0.5, 1, 2, 4 and 8 kHz, air and bone conduction	Hearing thresholds were higher in the XFS group than in the control group at 4 kHz (*p* = 0.004) and even higher at 8 kHz (*p* = 0.001), but not at frequencies of 0.25 (*p* = 0.316), 0.5 (*p* = 0.267), 1 (*p* = 0.082) and 2 (*p* = 0.131) kHz
			22 (44)	74.7 ± 7.78	40.9%		
Papadopoulos T.A. (2012) [70]	XFS	case-control	47 (94)	74.7 ± 6.78	49.0%	PTA 0.25, 0.5, 1, 2, 4 and 8 kHz air and bone conduction	Study group subjects displayed more severe sensorineural hearing loss (98%) compared to control group (86%) subjects at high frequencies of 4 and 8 kHz (*p* < 0.001), but not at low and medium frequencies of 0.25, 0.5 (*p* = 0.070), 1 and 2 kHz (*p* = 0.007)
			22 (44)	74.7 ± 7.78	41%		
Samarai V. (2012) [77]	XFS, XFG	prospective	50 (100)	60.73	46%	PTA 0.5, 1, 2, 3 kHz (speech comprehension), ISO 7029	SNHL was more common in the study group than in the control group (*p* = 0.001). 42% of patients in the study group had a higher HTL than the ISO 7029 median AAHL at 1, 2 and 3 kHz, compared to 24% in the control group; no difference in HL between XFS (*p* = 0.118) and XFG (*p* = 0.193).
			/	/	/		
Šarenac-Vulović T. (2014) [78]	XFS, XFG	cross-sectional	20 (n/a) XFG, 20 (n/a) XFS	73.41 ± 6.54 (XFS), 77.2 ± 3.9 (XFG)	25% XFS 30% XFG	PTA from medical history	HL higher in XFS (55%) and XFG (75%) (*p* = 0.033) than in controls (10%)
			20 (n/a)	63.4 ± 4.2	45%		
Shazly T.A. (2011) [79]	XFS	retrospective	320 (n/a)	68.15 ± 8.16	58.75%	medical history of HL	8.1% of XFS had HL vs. 2.3% in non-XFS (*p* < 0.001)
			/	/	/		
Singham N.V. (2014) [63]	XFS, XFG	case-control	68 (136)	68.5 ± 7.8	36.77%	PTA 0.25, 0.5, 1 and kHz	Higher hearing thresholds at 0.5, 1 and 2 kHz in XFS patients than controls (*p* = 0.01); no difference between right and left ear (*p* = 0.46).
			55 (110)	66.3 ± 7.4	41.7%		
Tekin S. (2021) [64]	XFS	case-control	40 (80)	67.13 ± 8.6	50%	PTA air (0.25, 5, 1, 2, 4, 6, kHz) and bone (0.5, 1, 2, 4 kHz) conduction.	PEX group: higher HTL in both air and both conductions compared to controls at 0.25, 0.5, 1, 2, 4 kHz (*p* ≤ 0.036), but not at 6, 8 kHz (*p* ≥ 0.151).
			46 (92)	64.04 ± 10.58	48%		
Temporale H. (2016) [62]	XFS	case-control	28 (56)	77.5 ± 7.6	32.1	PTA 0.5, 1, 2, 4 kHz, impedance audiometry, DPOAE and ABR	HTL significantly higher in PEX for 2 kHz (*p* = 0.021). In impedance audiometry tests, the stapedius reflex was identified in a greater proportion of patients in the XFS group than in the control group in all frequency ranges. No difference between the XFS group and the control group in the results of the DPOAE and ABR tests (*p* > 0.05).
			23 (46)	77.7 ± 8.8	21.7		
Tryggvason G. (2016) [72]	XFS, XFG, POAG	case-control	95 (190) POAG 75 (150) XFG 83 (166) XFS	77.4 ± 5.2 (XFS, XFG) 77.9 ± 5.2 (POAG)	30.4% (XFS, XFG) 35.8% (POAG)	PTA 0.5, 1, 2, 3, 4, 6 and 8 kHz, low and middle frequencies (PTA512—mean of thresholds at 0.5, 1 and 2 kHz) and high frequencies (PTA3468—mean of thresholds at 3, 4, 6 and 8 kHz), air conduction, tympanometry	No significant association between cases (XFS, XFG and POAG group) and controls (*p* < 0.05).
			123 (246)	76.8 ± 4.6	46.3%		
Turacli M.E. (2007) [57]	XFS, XFG	case-control	51 (102)	67.5	n/a	PTA 0.25, 0.5, 1, 2, 3, 4 and 6 kHz, bilateral or unilateral XFS	HL higher in XFS (66.7%) than controls (38.6%), no significant correlation with laterality of XFS and HL
			22 (44)	61	n/a		
Yazdani S. (2008) [47]	XFS, XFG	case-control	83 (166)	70.1 ± 7.7	72.3%	PTA 1, 2, 3 kHz, bilateral or unilateral XFS	HL in 88.4% ears in the XFS group vs. 53.6% in the control group without XFS (*p <* 0.001). The presence of glaucoma was not associated with higher HL both in the XFS group (*p* = 0.65) and in the control group without XFS (*p* = 0.48)
			83 (166)	69.8 ± 7.5	72.3%		
Yildirim N. (2017) [80]	XFS, XFG	case-control	100 (n/a)	69.1 ± 9.9	47.0%	HL%, not specified	HL was 5.4% in non-XFS participants and 34.0% in XFS patients (*p* < 0.001). 31. out of 34 XFS patients had SNHL, which was mild in 24 cases and moderate in 7.
			1909 (n/a)	59.2 ± 10.9	46.3%		
Zojaji R. (2011) [73]	XFS	case-control	33 (66)	72.2 ± 7.3	69.7%	PTA 0.25, 0.5, 1, 2, 3, 4 and 6 KHz	SNHL: 75.2% in the XFS group and 40% in the control group (*p* < 0.001); no significant difference between XFS and XFG (*p* = 0.768) and laterality of XFS and HL (*p* = 0.847).
			33 (66)	72.8 ± 6.1	63.6%		

AAHL1,2,3 = age-associated hearing loss summed over 1, 2 and 3 kHz; ABR = auditory brainstem response tests; APSA = antiphosphatidylserine antibodies; DPOAE = distortion product otoacoustic emission tests; HL = hearing loss; HTL = hearing thresholds; ISO 1964 = International Organization for Standardization 1964; ISO 7029 = International Organization for Standardization 7029; n/a = not available information; PTA = pure-tone audiometry; SNHL = sensorineural hearing loss; VEMP= vestibular-evoked myogenic potential; XFG = pseudoexfoliative glaucoma; XFS = pseudoexfoliation syndrome.

#### 3.1.2. Pseudoexfoliative Glaucoma

Twelve studies in our SRL analysed the relationship between XFG and HL. The relationship between XFG and hearing loss is more complex to elucidate, since most studies were designed to include XFS patients regardless of the presence of glaucoma, and most of the results on XFG refers to subgroup analyses. It is therefore difficult to ascertain the impact of pseudoexfoliation, glaucoma or their simultaneous presence in the association with hearing loss.

Most studies did not find significant differences in prevalence and level of hearing loss between XFS and XFG, despite nonsignificant increased frequency and higher hearing thresholds being reported in XFG patients. Turacli et al. detected SNHL in the majority of patients with XFS or XFG (66.7% vs. 38.6% in controls, *p* < 0.001) [57]; although hearing loss seemed to be more frequent in XFG (70.2%) compared to XFS without glaucoma (50%), the statistical significance of their difference was not tested. Likewise, Ozkan et al. [69]. did not find a relationship between SNHL and the degree of glaucomatous damage in the XFS group, although the prevalence of SNHL seemed higher in XFG (73.8% vs. 63.6% in XFS without glaucoma). Yazdani et al. also tested hearing function in 83 XFS/XFG patients, showing high prevalence of SNHL at 1, 2 and 3 kHz (88.4% of ears vs. 53.6% in age–sex matched controls, *p* < 0.001), however, glaucoma did not seem to associate with hearing loss; specifically, 95.3% of XFG patients (n = 43) and 92.5% of XFS without glaucoma (n = 40) suffered from hearing loss in at least one ear (*p* = 0.65) [47]. Similarly, Samarai et al. detected frequent SNHL at 1, 2 and 3 kHz in a mixed group of XFS/XFG patients (*p* < 0.05 vs. ISO 7029 age–sex matched controls), but, although SNHL prevalence being more common in XFG (66.7% vs. 38.6% of XFS without glaucoma) and mean hearing thresholds being higher (59.15 dB vs. 40.9 dB), this difference was not significant [77].

In 2014, Sarenac-Vulovic et al., in a cross-sectional study on 60 patients, included XFS, XFG and controls. A higher prevalence of hearing loss was found in XFG patients (75%), followed by XFS (55%) and controls (10%) [78]. Although a significant difference was detected between pseudoexfoliation groups and controls (*p* = 0.033), the difference between XFS and XFG was not significant [78]. It is worth noting that age distribution in their cohort of patients was significantly different, with XFG patients being older (mean 77.2 ± 3.9 years), followed by XFS (73.4 ± 6.5 years) and controls (63.4 ± 4.2 years) (*p* = 0.029) [78].

Definitively, no difference based on the presence of glaucoma was detected by Zojaji et al. and Cahill et al., despite confirming a positive association between XFS and HL [46,73].

The only study showing significant differences between XFS and XFG was conducted by Detorakis et al. XFG patients had significantly higher audiometric hearing thresholds for frequencies of 3 and 8 kHz in air conduction compared to XFS without glaucoma [74]. Respective differences were not statistically significant at 0.25 kHz, 1 kHz and 2 kHz in air conduction and for all examined frequencies in bone conduction [74]. The air–bone gap was also significantly higher in XFG patients for the 3 kHz frequency. Conversely, among controls, differences between patients with and without glaucoma were not significant. Additionally, tympanometric peak values were significantly lower in XFS/XFG compared with controls (0.66 ± 0.60 mL and 0.97 ± 0.60 mL, respectively; *p* = 0.004), but no differences in tympanometric peak values between glaucomatous and non-glaucomatous patients were shown in either group [74].

To elucidate the relationship between XFG, POAG and hearing loss, hearing abnormalities in glaucoma patients with XFG and without pseudoexfoliation were evaluated by Paliobei et al. and compared to norms provided by the ISO 7029 standard [76]. One hundred and ten patients with XFG and 85 POAG were investigated with PTA, DPOAE and ABRs to evaluate the function of peripheral and central auditory pathways, thus differentiating between pathologies at cochlear or retro-cochlear level. Both glaucoma types showed hearing loss at most tested frequencies, being far more prevalent in XFG. DPOAE amplitudes at high frequencies were reduced in both forms of glaucoma [76].

XFG patients had 4.34 times higher odds of showing pathologic ABR central transmission time (interpeak latencies I-III, I-V, wave V) compared to POAG (*p* < 0.001) [76]. Association between XFG and pathologic ARB remained significant after adjusting for sex, age and controlling for systemic factors (arterial pressure, coronary heart disease, cholesterol and stroke history), supporting the theory of the presence of a retrocochlear causative factor at the brainstem level. These results suggest that central auditory pathways might be more affected in XFG [76].

More recently, Gülyeşil et al. compared HL in XFG, POAG and age–sex matched controls. XFG patients had significantly higher hearing thresholds at 0.25, 0.5, 1, 2, 4, 8 and 10 kHz (*p* < 0.05) compared to controls and higher hearing threshold at 8 kHz compared to the POAG group (*p* = 0.002) [75].

Results from the above-mentioned studies suggest either that XFS, rather than XFG, is more likely to be primary associated with hearing loss or that the strength of the association of XFS with hearing function override its potential association with XFG.

### 3.2. Primary Open Angle Glaucoma and Hearing Loss

Eight articles in our SRL analysed the relationship between POAG and HL. Studies on POAG and SNHL showed that they share a similar pathophysiology of neurodegeneration [30,31]. It has been suggested that people with glaucoma are more vulnerable to central nervous system (CNS) neural decline, therefore they may also be more likely to reveal functional deficits beyond the visual system.

O’Hare et al. examined the auditory and visual temporal processing pathway of PAOG subjects [81]. A considerable number of people with POAG exhibited reduced ability to discern low-frequency sounds and understand speech compared to the control group [81]. More specifically, 36% of POAG participants experienced difficulty in differentiating low-frequency sounds, falling beyond the 90th percentile (*p* = 0.029), while 25% had speech perception scores below the lower limit (90th percentile) of the control range (*p* = 0.029). Visual speed discrimination of slow velocities was found to be outside the lower limit (90th percentile) of the range of controls in a significant proportion (39.13%) of POAG patients (*p* = 0.029). These findings indicate that some individuals with POAG may have an increased central nervous system vulnerability to damage, leading to auditory and visual processing dysfunction [81].

POAG and SNHL also share common risk factors, older age being the most important one. Hypertriglyceridemia can lead to vascular disfunction and can be implicated in the dysregulation of blood vessels supplying the optic nerve and the surrounding retinal tissue. The study by Kim et al. on the Korean population showed that aging and an increase in triglyceride level were independent risk factors for the simultaneous occurrence of POAG and hearing impairment [29]. They found that the weighted prevalence of POAG in patients with HL was higher (7.5%) than in patients without HL (3.2%), showing however, a non-significant difference [29].

As previously mentioned, Gülyeşil FF et al. compared HL in the POAG, XFG and control groups, indicating that hearing thresholds were higher in the POAG and XFG groups compared to control group [75]. In particular, POAG patients had significantly higher hearing thresholds at 0.5 kHz (*p* = 0.011) and 1 kHz (*p* = 0.003) compared to the control group [75]. This study showed a considerably higher probability of SNHL in POAG and XFG patients compared to controls, suggesting the need of routine otolaryngology examinations in older patients with POAG and XFG [75].

Interestingly, Neacșu et al. outlined a relationship between audiometry findings and ophthalmological parameters, observing an indirect relationship between MD in visual field examination and PTA results [82]. Compared to the control group, patients with POAG showed average levels of the PTA and modified visual field (VF) parameters. Multivariate analysis demonstrated that the correlation of PTA was indirect and reduced in intensity, both with MD (mean deviation in VF) (r = −0.108; *p* = 0.585), Cal HOV (Central height of the visual field) (r = −0.268; *p* = 0.168) and the slope profile of the right eye. The left ear PTA correlation was indirect, moderate in intensity, statistically significant with both MD (r = −0.584; *p* = 0.001) and slope profile (r = −0.377; *p* = 0.048) and reduced in intensity with Cal HOV (r = −0.147; *p* = 0.456) of the left eye. Therefore, changes in audiometry in POAG patients were in connection with ophthalmological parameters, suggesting that the auditory system could be affected in POAG [82].

A positive association between POAG and HL was highlighted in all articles examined, with the exception of the study from Hayreh SS et al., which found no association between optic nerve head ischemic disorders (including NTG, POAG and other types of glaucoma) and HL, suggesting that the two represent independent and unrelated disorders; age was the only significant common risk factor identified (*p* < 0.001) [83]. Also, Tryggvason G. et al. found no significant difference in HL between cases (XSF, XFG and POAG) and controls (*p* < 0.05) [72].

Details of the above-mentioned studies are presented in Table 2.

**Table 2:** Primary open angle glaucoma and hearing loss.

First Author (Year)	Glaucoma Type	Study Design	N Cases (n Ears)	Mean Age Patients (Mean ± SD)	% Males Patients	Outcome Measured	Main Findings
			N Controls (n Ears)	Mean Age Controls (Mean ± SD)	% Males Controls		
Chien H.W. (2019) [84]	POAG, NTG, ACG	Retrospective cohort study	15,686 SNHL	n/a	54.06%	HL based on medical history	Higher incidence rate of glaucoma in patients with SNHL (43.36 per 100,000 person–months) than in control group (32.93 per 100,000 person–months). NTG (*p* = < 0.0001) and ACG (*p* = 0.0148) > POAG (*p* = 0.1271).
			/	n/a	54.22%		
Gülyeşil F.F. (2023) [75]	POAG XFG	Case-control	24 (24) POAG 22 (22) XFG	64.50 ± 7 POAG 66.90 ± 4.51 XFG	50% POAG 50% XFG	PTA 0.25, 0.5, 1, 2, 4, 8, 1 kHz	Compared to controls: POAG: higher hearing thresholds at 0.5 (*p* = 0.011) and 1 kHz (*p* = 0.003). XFG: higher hearing thresholds at 0.25 (*p* = 0.009), 0.5 (*p* = 0.009), 1 (*p* = 0.001), 2 (*p* = 0.005), 4 (*p* = 0.001), 8 (*p* = 0.010) and 10 kHz (*p* = 0.009); XFG group: higher hearing threshold at 8 kHz than the POAG group (*p* = 0.002).
			21 (21)	64.38 ± 4.36	57.1%		
Hayreh S.S. (1999) [83]	NTG, POAG, other glaucoma types	Prospective cohort study	36 NTG 138 POAG	69.8± 14.6 NTG, 69.8 ± 13.7 POAG	25% NTG, 51% POAG	HL based on medical history/patients’ interview	No association between glaucoma and HL. Only association of HL with age (*p* < 0.001).
			/	/	/		
Kim J.M. (2020) [29]	POAG	Cross-sectional	236 (472) POAG 51 (102) POAG AND HL 62 (124) HL	48.5 ± 1 POAG 66.9 ± 1.8 POAG AND HL 62.6 ± 0.6 HL	55.4% POAG 68.2% POAG AND HL 58.3% HL	0.5, 1, 2, 3, 4 and 6 kHz	Higher glaucoma prevalence (7.5%) in patients with HL (PTA > 40 dB) than in patients without HL (3.2%). Glaucoma was significantly associated with HL (odds ratio, 3120; 95% confidence interval, 2.25–4.32).
			941 (1882)	41.2 ±0.2	48.4%		
Neacșu A.M. (2023) [82]	POAG	prospective	16 (32)	63.69	31.3%	PTA 0.125, 0.25, 0.5, 1, 2, 4 and 8 kHz; MD, Cal HOV	Correlation of PTA was indirect, reduced in intensity, both with MD (r = −0.108; *p* = 0.585), Cal HOV (r = −0.268; *p* = 0.168) and the slope profile of the right eye.
			12 (24)	58.92	25%		
O’Hare F. (2012) [81]	POAG	Case-control	25 (50)	n/a	n/a	Auditory low-frequency discrimination, speech perception, visual speed discrimination, visual global motion detection, auditory amplitude modulation detection and auditory frequency discrimination (PTA 0.25, 0.5, 1, 2, 3, 4, 5, 6 kHz)	36% of POAG participants showed impaired low-frequency discrimination (*p* = 0.028): (POAG: 14.6 ± 7.1 Hz; control: 10.5 ± 3.5 Hz) 25% of POAG patients had speech perception scores outside the lower limit of the control range (*p* = 0.029) 39.13% of POAG patients had results outside the lower limit (90th percentile) of the range in control performance (*p* = 0.029) for slow speed visual discrimination.
			25 (50)	n/a	n/a		
Paliobei V.P. (2011) [76]	XFG, POAG	prospective	85 (170) POAG 110 (220) XFG	67.4 ± 4.6 XFG 64.8 ± 6.5 POAG	44.7% POAG 58.2% XFG	PTA (0.25, 0.5, 1, 2, 4 and 8 kHz), audiometry, tympanometry, stapedial reflex test, ABRs, DPOAEs, ISO 7029	HL was more prevalent with POAG and XFG compared to the ISO 7029 (XFG > POAG); DPOAs amplitudes reduced in both POAG and XFG. Pathologic ABR was 4 times higher in XFG than POAG.
			/	/	/		
Tryggvason G. (2016) [72]	XFS, XFG, POAG	Case-control	95 (190) POAG 75 (150) XFG 83 (166) XFS	77.4 ± 5.2 (XFS, XFG) 77.9 ± 5.2 (POAG)	30.4% (XFS, XFG) 35.8% (POAG)	PTA 0.5, 1, 2, 3, 4, 6 and 8 kHz, low and middle frequencies (PTA512—mean of thresholds at 0.5, 1 and 2 kHz) and high frequencies (PTA3468—mean of thresholds at 3, 4, 6 and 8 kHz), air conduction, tympanometry	No significant difference in HL between cases (XSF, XFG and POAG) and controls (*p* < 0.05)
			123 (246)	76.8 ± 4.6	46.3%		

ABRs = auditory brainstem response; ACG = angle-closure glaucoma; Cal HOV = central height of the visual field; DPOAEs = distortion product otoacoustic emissions; ISO 7029 = International Organization for Standardization 7029; MD = mean deviation; NTG = normal tension glaucoma; POAG = primary open-angle glaucoma; PTA = pure tone audiometry; XFG = pseudoexfoliative glaucoma; XFS = pseudoexfoliation syndrome.

### 3.3. Normal Tension Glaucoma and Hearing Loss

Four articles in our SLR examined NTG and HL, almost all showing positive association, except in one case. Patients with normal tension glaucoma showed disease progression without measured IOP elevation. For this reason, other risk factors have been implicated in the pathogenesis of NTG, such as cardiovascular and haematological risk factors as well as genetic and immunological aspects [85,86,87,88,89,90].

Regarding the potential correlation between NTG and progressive SNHL, some authors focused on the role of autoantibodies against antigens in the inner ear [91,92].

Both Kremmer S. and Bachor E. evaluated the role of autoimmunity in NTG and SNHL by measuring the serum levels of antiphosphatidylserine antibodies (APSA), one of the hallmarks of the antiphospholipid syndrome [91,92]. Kremmer et al. showed that patients with NTG had significantly higher concentrations of IgG APSA compared to controls (*p* < 0.05), and elevated APSA concentrations showed significantly higher concentrations in NTG with progressive SNHL compared to NTG patients with normal hearing (*p*< 0.01) [92]. Significantly higher concentrations of IgG APSA in NTG with progressive SNHL compared to normal hearing and controls were also found by Bachor et al., who additionally found the concentrations of IgM APSA were significantly elevated in all subgroups of NTG patients, as well as in in NTG patients with normoacusis, compared to controls [91]. The exact mechanisms underlying the association between APSA levels and NTG are not fully understood; however, higher levels of APSA in patients with NTG could be indicative of an underlying autoimmune or vascular mechanism contributing to the pathogenesis of NTG. According to their hypothesis, APSA may be involved in chronic injuries to the vascular endothelial cells, resulting in thromboembolism of small vessels and finally leading to disturbances of microcirculation in the inner ear and eye, thereby explaining both hearing loss and glaucoma [91].

Chien H.W. et al. performed a retrospective, population-based cohort study to investigate the incidence of POAG, NTG and ACG in patients with SNHL over a 16-year follow-up [84]. They demonstrated that individuals with SNHL had significantly higher chances of developing glaucoma compared to controls. Specifically, the incidence rate of glaucoma in the study group was 43.36 per 100,000 person–months, while being 32.93 per 100,000 person–months in controls. Moreover, the increased incidence was more evident for NTG (*p* = < 0.0001) and ACG (*p* = 0.0148), rather than for POAG (*p* = 0.1271).

In contrast, as previously described analysing the relationship between POAG and SNHL, Hayreh et al. did not find any association between optic nerve head ischemic disorders including NTG, POAG and other glaucoma types and HL, with age being the only factor determining significant association [83].

Details of the above-mentioned studies are presented in Table 3.

**Table 3:** Normal tension glaucoma and hearing loss.

First Author (Year)	Glaucoma Type	Study Design	N Cases (n Ears)	Mean Age Patients (Mean ± SD)	% Males Patients	Outcome Measured	Main Findings
			N Controls (n Ears)	Mean Age Controls (Mean ± SD)	% Males Controls		
Bachor E. (2005) [91]	NTG	prospective	34 (n/a)	65	32.3%	APSA, audiograms, stapedial thresholds, otoacoustic emissions, positional and caloric testing	11/34 NTG patients had SNHL; NTG and progressive SNHL showed higher APSA IgG compared to patients with normal hearing NTG and controls (*p*< 0.01). IgM APSA were higher in all NTG patients (*p* < 0.05).
			40 (n/a)	62	/		
Chien H.W. (2019) [84]	POAG, NTG, ACG	Retrospective cohort study	15686 SNHL	n/a	54.06%	HL based on medical history	Higher incidence rate of glaucoma in patients with SNHL (43.36 per 100,000 person–months) than in control group (32.93 per 100,000 person–months). NTG (*p* = < 0.0001) and ACG (*p* = 0.0148) > POAG (*p* = 0.1271).
			/	n/a	54.22%		
Hayreh S.S. (1999) [83]	NTG, POAG, other glaucoma types	Prospective cohort study	36 NTG 138 POAG	69.8 ± 14.6 NTG, 69.8 ± 13.7 POAG	25% NTG, 51% POAG	HL based on medical history/patients’ interview	No association between glaucoma and HL. Only association of HL with age (*p* < 0.001).
			/	/	/		
Kremmer S. (2004) [92]	NTG	Cross-sectional	34	65	32.3%	PTA, stapedial thresholds and transitory otoacoustic emissions, APSA	68% of NTG pt had HL; excluding presbiacusis (35%), 32% had HL, defined by age-mached controls. APSA concentrations were significantly higher in NTG compared to controls regardless of HL (*p* < 0.05). APSA were higher in patients with NTG and HL than iin NTG and normoacusis.
			/	/	/		

ACG = angle-closure glaucoma; APSA = antiphosphatidylserine antibodies; HL = hearing loss; n/a = not available information; NTG = normal-tension glaucoma; POAG = primary open-angle glaucoma; PTA = pure tone audiometry; SNHL = sensorineural hearing loss.

### 3.4. Angle Closure Glaucoma

Only the retrospective, population-based cohort study by Chien et al. analysed the relationship between ACG and HL, showing higher incidence of ACG in patients with SNHL compared to normal hearing controls. Interestingly, the overall increased glaucoma incidence in the SNHL group was higher for ACG compared to POAG (Table 3) [84].

## 4. Discussion

The aim of our systematic review was to evaluate the relationship between glaucoma, pseudoexfoliation and HL. Most studies in the literature concerned the relationship between XFS and HL, although several types of glaucoma were also analysed in relation to the presence of HL.

The association between XFS and HL seemed to be confirmed in the majority of studies. The shared embryological origins from the neural ectoderm of the ocular anterior segment and the basilar and tectorial membrane of the inner ear provide a possible explanation for this association [93]. The presence of pseudoexfoliation fibres in multiple extra-ocular tissues suggests that there may be widespread systemic involvement in addition to the ocular manifestations. This indicates that the association between XFS and HL may be part of a larger systemic condition rather than an isolated phenomenon [24]. It seems plausible that the fibrillar-like protein material that accumulates on the anterior lens capsule and in the trabecular meshwork can also accumulate in the tectorial and basilar membranes and stria vascularis in the inner ear, therefore damaging the inner ear structures [46,57]. Additionally, the association between XFS and HL might also have a cardiovascular origin. In fact, the presence of exfoliative material in the walls of arteries may lead to problems with the inner lining and inflammation, increasing the likelihood of cardiovascular events [94]. Similarly, cardiovascular disease is commonly thought to cause HL due to compromised blood flow to the cochlea. The reduction may be due to microvascular changes in the stria vascularis or to macrovascular changes of the internal auditory artery [95]. Lastly, both XFS and HL share a common neurodegenerative pathogenesis. Studies examining the relationship between XFS and neurodegenerative diseases found that the accumulation of protein aggregates, similar to those seen in XFS, may also be involved in the pathogenesis of neurodegenerative disorders. The presence of α-synuclein in the ocular tissues of individuals with XFS suggests a possible connection between the two conditions, warranting further investigation into the shared mechanisms and potential therapeutic targets [96]. HL, likewise, can be caused by neurodegenerative conditions, affecting the central auditory system and leading to significant and early impairment in hearing function [97].

It is however unclear whether the presence of XFG is further associated with SNHL, mostly due to the lack of studies specifically designed to answer this question. In fact, most studies compared mixed populations of XFS and XFG patients to controls and results on XFG mainly referred to post hoc analyses. Given the strength of the association between XFS and HL; although several studies detected higher prevalence of HL in XFG compared to XFS, this difference was often non-significant. It remains therefore unclear whether XFS, rather than glaucoma, is more likely to be associated with hearing loss or the strong association between XFS and HL can mask the significance of the association with XFG.

With regard to PAOG and HL, the majority of articles confirmed their association. Recognized risk factors for both diseases were age and hypertriglyceridemia [83]. It is well known that elevated triglyceride levels contribute to vascular and metabolic conditions that may affect blood flow and tissues health, including eyes and ears. High levels of triglycerides can lead to atherosclerosis and reduced blood flow, which may in turn contribute to optic nerve damage in glaucoma and inner ear damage in HL. Adequate blood flow is essential for the health of the optic nerve and the cochlea, thus microvascular changes could feasibly raise the risk for both SNHL and glaucoma. Age was also identified as a common risk factor for POAG and HL [83]. Mechanisms behind this correlation are not fully understood, but age-related changes in the eye can include increased rigidity of the eye’s drainage system, leading to an increase in intraocular pressure, which can damage the optic nerve. Regarding its correlation to HL, aging leads to damage to the hair cells in the cochlea, leading to decreased speech discrimination and perception [83].

Two articles described the bidirectional association between POAG and hearing loss [65,84]. It is interesting to underline that not only was the prevalence of HL was higher in patients with POAG, but HL was related to an increased incidence of POAG. However, the relationship between POAG and HL may be multifactorial and influenced by various systemic and genetic factors that may contribute to the vulnerability of both sensory systems. Further research specifically targeted to assess the correlation between POAG and HL is warranted to provide a more comprehensive understanding of this association.

Research also showed that sensorineural hearing loss is more prevalent in patients with NTG compared to healthy subjects. As this condition is unrelated to high IOP levels, risk factors other than IOP are thought to be implicated in the development of NTG. The greater prevalence of NTG in patients with SNHL suggested that the overall higher glaucoma incidence in patients with SNHL might depend on the generalized vulnerability of the nervous system rather than the elevated intraocular pressure [84]. The role of autoimmunity in the development of HL in patients with NTG was also analysed and, in particular, APSA antibodies serum levels were found to be higher in patients with both NTG and HL, suggesting that autoimmunity could represent a potential common risk factor [91,92]. APSA have been associated with thrombosis, which can affect circulation. In the ear, thrombosis could cause ischemia in the labyrinth, leading to HL. NTG has also been considered as a vascular issue where optic nerve damage may occur due to insufficient blood supply despite normal eye pressure. If this correlation were to be confirmed by further studies, the presence of autoimmune factors could open avenues for immunomodulatory treatments [91,92]. The recognition of this association could prompt clinicians to monitor for the presence of antiphospholipid antibodies in patients presenting with either NTG or SNHL. Understanding the shared mechanisms of NTG and SNHL might lead to new treatment approaches, possibly involving immunomodulatory or anticoagulative treatments that target the autoimmune response or improve vascular health. Early detection and treatment could potentially slow the progression of both conditions [91,92]. Moreover, managing risk factors related to blood circulation and thrombosis could be beneficial for both NTG and PSHL. However, further research is needed to confirm this correlation and to understand the underlying mechanisms.

The relationship between XFS, glaucoma and HL is still a topic of debate, and further research is needed to fully understand their connection. It is important to consider the potential systemic and genetic factors that may influence this relationship. By conducting targeted studies to assess the correlation between XFS, glaucoma and HL, researchers can gain valuable insights that may contribute to more effective management and treatment approaches for individuals affected by these conditions. Additionally, understanding the underlying mechanisms of this association could lead to advancements in both ophthalmology and otology, ultimately benefiting patient care and outcomes.

## 5. Conclusions

The association between pseudoexfoliation, glaucoma and hearing loss is not yet fully understood, and more studies are required to elucidate the underlying common pathogenetic mechanisms. Nevertheless, both glaucoma and HL are prevalent and chronic conditions that affect quality of life in the elderly, and most studies in our SLR suggested their association [75]. Understanding the relationship between these two conditions could potentially lead to more targeted and effective management of individuals with XFS, glaucoma and comorbid sensorineural hearing loss. Collaboration between ophthalmologists and otolaryngologists may therefore be beneficial for an early detection of the associated disease and for providing better comprehensive care for patients. A routine hearing exam has been suggested in glaucoma patients for timely intervention and care of hearing loss [75] and dilated fundus exam may be suggested in individuals with SNHL to improve early glaucoma detection.

## Data Availability

Not applicable.
